# Specific questionnaire detects a high incidence of intra-operative hypersensitivity reactions

**DOI:** 10.6061/clinics/2018/e287

**Published:** 2018-05-08

**Authors:** Laila S. Garro, Marcelo V. Aun, Iracy Sílvia C. Soares, Marisa R. Ribeiro, Antônio A. Motta, Jorge Kalil, Mariana C. Castells, Maria José C. Carmona, Pedro Giavina-Bianchi

**Affiliations:** I Divisao de Imunologia Clinica e Alergia, Faculdade de Medicina (FMUSP), Universidade de Sao Paulo, Sao Paulo, SP, BR; II Departamento de Anestesiologia, Faculdade de Medicina (FMUSP), Universidade de Sao Paulo, Sao Paulo, SP, BR; III Division of Rheumatology, Immunology and Allergy, Department of Medicine, Brigham and Women’s Hospital, Harvard Medical School, Boston, Massachusetts, USA

**Keywords:** Anaphylaxis, Anesthesia, Drug Allergy, Intraoperative, Questionnaire

## Abstract

**OBJECTIVE:**

To assess the incidence of intra-operative immediate hypersensitivity reactions and anaphylaxis.

**METHODS:**

A cross-sectional observational study was conducted at the Department of Anesthesiology, University of São Paulo School of Medicine, Hospital das Clínicas, São Paulo, Brazil, from January to December 2010. We developed a specific questionnaire to be completed by anesthesiologists. This tool included questions about hypersensitivity reactions during anesthesia and provided treatments. We included patients with clinical signs compatible with immediate hypersensitivity reactions. Hhypersensitivity reactions were categorized according to severity (grades I-V). American Society of Anesthesiologists physical status classification (ASA 1-6) was analyzed and associated with the severity of hypersensitivity reactions.

**RESULTS:**

In 2010, 21,464 surgeries were performed under general anesthesia. Anesthesiologists answered questionnaires on 5,414 procedures (25.2%). Sixty cases of intra-operative hypersensitivity reactions were reported. The majority patients (45, 75%) had hypersensitivity reactions grade I reactions (incidence of 27.9:10,000). Fifteen patients (25%) had grade II, III or IV reactions (intra-operative anaphylaxis) (incidence of 7:10,000). No patients had grade V reactions. Thirty patients (50%) were classified as ASA 1. The frequency of cardiovascular shock was higher in patients classified as ASA 3 than in patients classified as ASA 1 or ASA 2. Epinephrine was administered in 20% of patients with grade III hypersensitivity reactions and in 50% of patients with grade II hypersensitivity reactions.

**CONCLUSIONS:**

The majority of patients had hypersensitivity reactions grade I reactions; however, the incidence of intra-operative anaphylaxis was higher than that previously reported in the literature. Patients with ASA 3 had more severe anaphylaxis; however, the use of epinephrine was not prescribed in all of these cases. Allergists and anesthesiologists should implement preventive measures to reduce the occurrence of anaphylaxis.

## INTRODUCTION

According to the International Consensus (ICON) on Drug Allergy, hypersensitivity reactions (HSR) to drugs are adverse drug effects that clinically resemble allergic reactions. These reactions are classified as immediate or non-immediate (delayed) according to their onset during treatment 1. Immediate HSRs typically occur within 1 hour after last drug intake and include signs and symptoms such as urticaria, angioedema, rhinitis, conjunctivitis, bronchospasm, gastrointestinal symptoms (nausea, vomiting, diarrhea, or abdominal pain) and anaphylaxis 1. On the other hand, the ICON on Anaphylaxis defined anaphylaxis as a serious, generalized or systemic HSR that can be life-threatening or fatal 2. In general, anaphylactic reactions are defined as immediate HSRs that compromise two or more organs or systems.

In anesthesia, intra-operative anaphylaxis is a rare yet troublesome complication that can disrupt the surgical procedure and can ultimately be fatal 3-5. However, the incidence of milder, immediate HSRs and anaphylaxis during anesthesia remains unknown mainly because cases are not systematically reported in most countries. The incidence of intra-operative anaphylaxis can be reduced by preventing these reactions in patients with a history of previous reactions during surgical procedures 6. Early identification of the symptoms and signs of a reaction can prevent severe anaphylaxis.

In France, cases of intra-operative anaphylaxis have been reported since 1985, enabling an understanding of these reactions and the implementation of effective interventions to prevent them 6,7. After eight years of monitoring these cases, the incidence of intra-operative immediate HSR was estimated to range from 0.5 to 2.9:10,000 6-8. Worldwide, intra-operative systemic reactions account for 9-19% of all surgical complications and 5-7% of all deaths during anesthesia 7. Neuromuscular blocking agents (NMBAs) continue to be the main etiological agents (implicated in 58.08% of cases) and are followed by latex (in 19.65%) and antibiotics (in 12.85%) 8.

Analysis of anesthetic records provides essential information about intra-operative HSR, such as clinical manifestations, drugs that might be involved, and timing between the drug infusion and the onset of a reaction 5. Studies addressing intra-operative immediate HSR are important and can help prevent further adverse reactions. The aim of this study was to evaluate the incidence and profiles of intra-operative immediate systemic HSR and analyze potentially associated factors.

In this study, allergists developed a questionnaire, which was answered by anesthesiologists just after surgical procedures during the year 2010. With this new tool, we found a high incidence of immediate hypersensitivity reactions, from mild cutaneous reactions to anaphylaxis (7:10.000 anesthesias). This simple questionnaire can increase diagnosis of perioperative hyper- sensitivity reactions.

## MATERIALS AND METHODS

**Figure 1:**
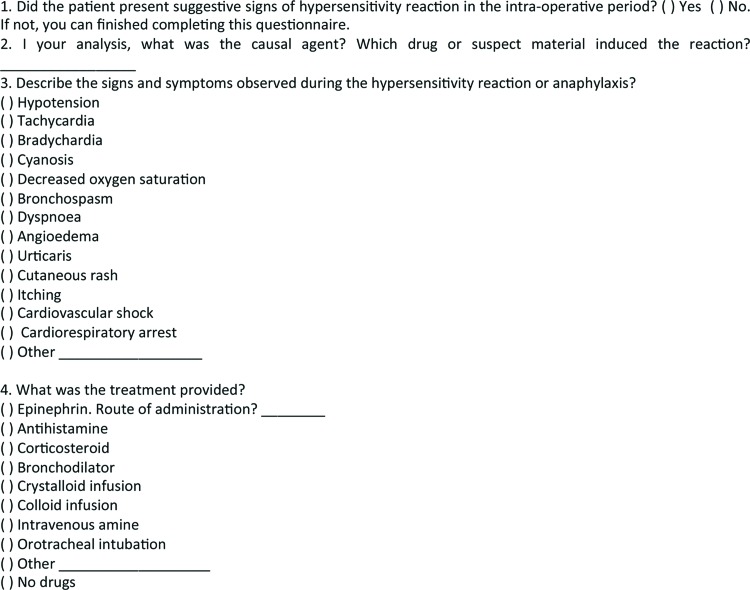
Specific questionnaire to identify intra-operative immediate hypersensitivity reactions.

This cross-sectional observational study was conducted using data provided by anesthesiologists through a voluntary-encouraged reporting system. Allergists with expertise in drug hypersensitivity, together with anesthesiologists, developed a specific questionnaire to assess the occurrence of systemic HSR during intra-operative periods (Figure 1). The questionnaire was attached to all medical records related to the anesthesia applied during surgical procedures performed from January to December 2010 at *Hospital das Clínicas*, São Paulo, Brazil. Anesthesiologists were encouraged to complete the questionnaire at the end of each surgical procedure whether or not intra-operative reactions occurred. Cases of intra-operative immediate HSR were selected for analysis. In addition to the data provided by the anesthesiologists on the questionnaires, we analyzed data obtained from the medical records, such as gender, age, types of surgical procedures associated with the reported HSR, and the American Society of Anesthesiologists (ASA) physical status classification (ASA 1-6) was used as an indicator of physical status 9.

Symptoms and signs reported by anesthesiologists in the questionnaire were assessed. We considered the following symptoms and signs to be compatible with immediate reactions: angioedema, urticaria, cutaneous rash, bronchospasm, dyspnea, decreased oxygen saturation, cyanosis, tachycardia, bradycardia, mild hypotension, cardiovascular shock, cardiorespiratory arrest, vomiting, nausea and diarrhea.

The severity of systemic reactions was classified in five stages of increasing severity according to international guidelines 7,10: grade I (generalized cutaneous signs: erythema, urticaria with or without angioedema), grade II (moderate multi-organ involvement with cutaneous signs, hypotension and severe tachycardia, bronchial hyperreactivity), grade III (severe life-threatening multi-organ involvement that requires specific treatment: collapse, tachycardia or bradycardia, cardiac arrhythmias, bronchospasm; cutaneous signs may be absent or occur only after arterial blood pressure recovers a normal value), grade IV (circulatory and/or respiratory arrest) or grade V (death due to inefficient cardiorespiratory resuscitation).

Provided treatments were also evaluated with special attention to the use of epinephrine.

We assessed drugs or materials mentioned by anesthesiologists as possible causative agents (NMBAs, opioids, antibiotics, non-steroidal anti-inflammatory drugs, local anesthetics, immunosuppressants, packed platelets, and other substances, such as latex, iodine solution and chlorhexidine), and we associated them with the severity of reactions.

### Ethics

This study was approved by the Research Ethics Committee of the University of São Paulo, School of Medicine, located in the city of São Paulo, Brazil (protocol number CAPPesq 0791/10).

### Statistical analysis

Categorical variables were compared using Fisher’s exact test, and *p*<0.05 was considered statistically significant.

## RESULTS

**Figure 2:**
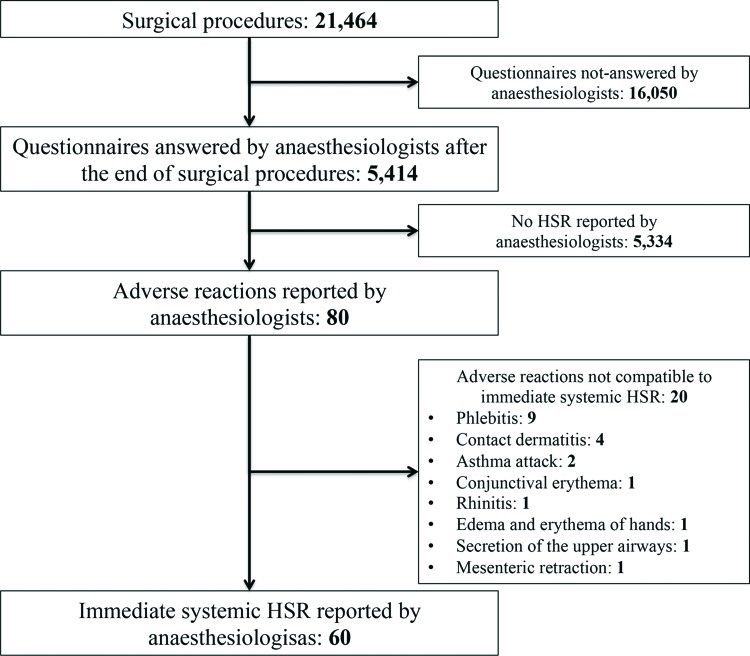
Case selection algorithm based on questionnaires answered by anesthesiologists about the occurrence of systemic hypersensitivity reactions (HSR) during intra-operative period in procedures performed from January to December 2010. HSR, hypersensitivity reactions.

During the study period (from January to December 2010), 21,464 surgical or diagnostic-therapeutic procedures were performed under general anesthesia. Anesthesiologists answered questionnaires on 5,414 procedures (25.2% of all procedures during the study period), and 60 cases of intra-operative immediate HSR were reported, as shown in Figure 2. If we assume that unanswered questionnaires represent the absence of systemic HSR, the incidence of HSR was 27.9:10,000.

We evaluated the 60 patients diagnosed with intra-operative systemic reactions, 34 (56.7%) of whom were female, and their mean age was 36.7 (±19.3) years. Non-vascular abdominal procedures, followed by plastic surgery and ophthalmological surgery, were the most commonly cited types of surgical procedures associated with systemic reactions (Table 1). Thirty patients (50%) had ASA physical status class 1, 20 patients (33.4%) had ASA 2 and 10 patients (16.7%) had ASA 3.

**Figure 3:**
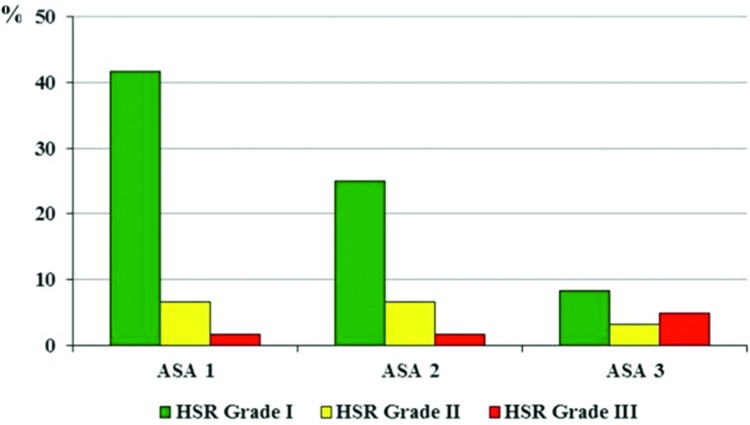
Hypersensitivity reaction grade of severity according to ASA physical status (n=60). HSR, hypersensitivity reactions.

Skin manifestations were the most commonly reported signs and symptoms, occurring in 57 (95%) patients, and they were mainly described as rashes. We found that 45/60 patients (75%) presented only cutaneous signs and were classified as grade I reactions according to international guidelines 5,8. The remaining 15 patients (25%) presented grade II (16.7%) and III (8.3%) systemic immediate HSR, which were considered anaphylactic reactions. No cardiac or respiratory arrests and no deaths were reported (grade IV and V, respectively). Then, we found an incidence of 7:10,000 for intra-operative anaphylaxis. The clinical features observed in all 60 intra-operative immediate HSR are described in Table 2. The frequency of cardiovascular shock was higher in patients with ASA physical status 3 than in those classified with ASA 1 or 2 (Figure 3).

Epinephrine was administered in 6 (50%) patients with grade II reactions and in 1 (20%) individual with a grade III reaction (*p*>0.05). Epinephrine was not used in all cases with compromised cardiorespiratory function. Two (50%) of the remaining 4 patients who presented grade III reactions were treated with other vasopressors. One patient was treated with a continuous infusion of intravenous norepinephrine and the other received metaraminol and terlipressin. No patients with cutaneous isolated signs (grade I reactions) were treated with epinephrine.

**Figure 4:**
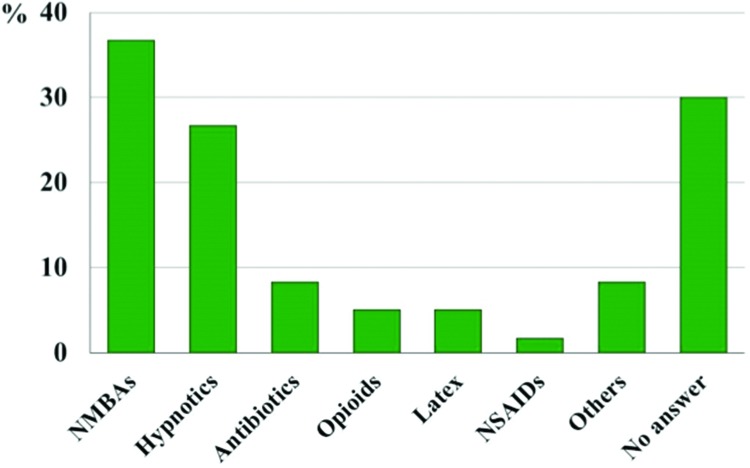
Possible causative agents of reaction as determined by anesthesiologists on the basis of identified clinical signs (n=60). NMBAS: Neuromuscular blocking agents, NSAIDS: Non-steroidal anti-inflammatory drugs.

The drugs most often judged to be the culprit agent of the systemic reaction were NMBAs, which were implicated in 36.7% of patients, followed by hypnotics in 26.7%. Anesthesiologists did not report a culprit agent in 30% of the 60 cases (Figure 4). There was no difference between agents suspected by anesthesiologists and the severity of reactions (Table 3).

## DISCUSSION

Within our study sample, the incidence of intra-operative anaphylaxis was 7 cases per 10,000 and the incidence of systemic immediate HSR, including isolated cutaneous reactions, was 27.9:10,000. This incidence of intra-operative anaphylaxis was higher than what has been reported in the literature 6-8. The incidence of intra-operative allergic and non-allergic anaphylactic reactions varies among countries and centers and across studies, depending on the applied methodology. In a recently published study from the Cleveland Clinic, approximately 15:10,000 immediate HSR were found during non-cardiac surgeries 11. They used a novel electronic search protocol developed to identify potential reactions that included the administration of epinephrine, antihistamines and blood tests, such as immunoglobulin E (IgE) and tryptase 11.

The use of a specific questionnaire aimed at anesthesiologists (even though its use was voluntarily) might have stimulated reporting and could therefore explain the relatively high incidence observed in this study. Milder cases were reported (grade I reactions) in the questionnaire that might not have been reported in regular conditions. The frequency of answered questionnaires was low (25.2%). We believe that unanswered questionnaires corresponded to surgeries with no adverse reactions. On the other hand, if we evaluated only answered questionnaires, our incidence of HSRs would be much higher and would have possibly made our data unreliable.

We did not find a large difference in the incidence of HSRs between genders. It has been postulated that sexual hormones are responsible for the fact that in adults, the incidence of intra-operative anaphylaxis is higher in women than in men, given that the incidence is similar between both genders before puberty 8. We did not evaluate children in this group of patients because the study was conducted at an adult university hospital; females accounted for approximately half of the study sample.

The most common surgical procedures in the 60 evaluated cases were non-vascular abdominal surgeries. This result might be related to the fact that these surgeries last longer than others, resulting in longer anesthesia times and higher doses of the employed drugs, and longer exposure times to latex for the mucous membranes. Moreover, half of the evaluated cases were ASA 1 physical status. At our hospital, the majority of surgeries are non-urgent and occur when the patient is clinically stable.

Skin reactions constituted the most common clinical manifestation of intra-operative reactions. In a study conducted in France involving 2,516 patients with intra-operative anaphylaxis, skin symptoms were found to be more common in cases of non-IgE-mediated anaphylaxis than in those of IgE-mediated anaphylaxis (in 95.3% *vs*. 70.2%) 8. Therefore, there can be cases of intra-operative anaphylaxis in which no skin reaction exists or in which the reaction is discreet and goes undiagnosed when it occurs in isolation. Consequently, it is extremely important that anesthesiologists pay careful attention to patient skin conditions because surgical drapes can obscure these visible signs. It is important to diagnose patients with milder reactions because these diagnoses might prevent more severe reactions. Despite the relatively high incidence of intra-operative anaphylaxis observed in the present study, no deaths were reported in 2010.

After the patients had been classified by ASA physical status and systemic immediate HSR severity, we identified an association between these two factors. We found no cardiovascular shock in patients with an ASA physical status of 1. Grade III reactions were mainly present in patients with ASA 3, showing that a higher frequency of comorbidities worsens the severity of anaphylaxis. In addition to treatment with beta-blockers or angiotensin-converting enzyme inhibitors, some comorbidities, such as uncontrolled asthma and severe cardiovascular disease, are associated with a higher risk of intra-operative anaphylaxis 10. Other risk factors for intra-operative immediate reactions have already been cited in the literature, such as a history of adverse reactions to anesthetics in previous surgeries, symptoms suggestive of latex allergy or allergy to fruits (possible cross-reactivity with latex) and children undergoing multiple surgeries. There is no evidence of increased risk in patients with atopy or allergy to drugs not used in anesthesia 7.

Based on the severity of the reactions, we assessed the use of epinephrine, indicated as the first-choice treatment when anaphylaxis appears, to impair cardiopulmonary function 12,13. In the present study, epinephrine was used in 7 patients (6 patients with grade II reactions and 1 patient with a grade III reaction). However, epinephrine was not used in the remaining 4 cases of grade III reactions; a lack of epinephrine is associated with a poor prognosis. Half of these patients were treated with other vasopressors, but the literature contains limited data on their use for anaphylaxis treatment. Late administration of epinephrine has been demonstrated as the main factor associated with death from anaphylaxis 14. We believe that anesthesiologists can misdiagnose anaphylaxis as hypotension during surgery, as these HSRs are uncommon, whereas there are many other causes of hypotension under anesthesia. These physicians probably need to be better trained in the management of anaphylactic reactions because diagnosis leads to treatment.

The surveyed anesthesiologists indicated which agent they suspected as the cause of the reaction. Therefore, the data related to the main groups of possible causative agents in this study refer to clinical assessments of the involved anesthesiologists. In agreement with other samples described in the literature 3,5,7,15, NMBAs were the most common agents implicated by the anesthesiologists. Nevertheless, after NMBAs, the second principal agents involved in HSRs were hypnotics; in contrast, other studies reported latex 3,5,7,15. In United States, antibiotics are the primary cause of intra-operative anaphylaxis 16. As the causative agents were defined by the clinical judgment of anesthesiologists, it is possible that these data might not be corroborated by *in vivo* and *in vitro* tests. Additional studies are needed to establish the main causative agents. In five cases, the clinical observations of anesthesiologists noted other possible causes of intra-operative reactions: local anesthetic, immunosuppressants, packed platelets, iodine solution and adhesive plaster.

We did not find any difference between agents implicated by the clinical judgment of anesthesiologists and the severity of the clinical manifestations. Another study showed that intra-operative anaphylaxis caused by NMBAs or antibiotics were more severe than those caused by latex 17. The authors demonstrated that the clinical variability of the reactions was also related to the involved pathophysiological mechanism. In that study, cardiovascular collapse and bronchospasm were found to be more common among patients with IgE-mediated HSR than among those with non-IgE-mediated HSR with the latter mainly developing skin symptoms 17. We recently demonstrated that drug-induced anaphylaxis was more severe than IgE-mediated anaphylaxis and that these results were independent of whether they were related to surgical procedures or not 18. We believe that the small number of reactions in our study made it difficult to show differences in the severity of reactions according to suspected drugs. Furthermore, only some of these patients were subjected to allergy investigation after their reaction (data not shown).

Reporting of intra-operative immediate HSR cases is important because it allows at-risk patients to be identified, thereby potentially preventing fatal reactions and increasing knowledge of the most common causative agents. Prevention measures, such as opting to use drugs that are less related to immediate HSR and early diagnosis of latex allergy, can be established through compulsory reporting and examinations of reported cases. Both approaches would reduce the number of cases of intra-operative anaphylaxis. In addition, the possibility of intra-operative reactions should considered when assessing the risk-benefit ratio of various anesthetic techniques 8.

The main limitation of the present study is that the clinical data and diagnoses were based on assessments made by anesthesiologists, who may not be used to these signs, particularly cutaneous reactions. Moreover, they did not have access to serum tryptase measurements, which are an important diagnostic test for systemic reactions.

Collaboration between anesthesiologists and allergists is essential for guiding investigations conducted by the latter 7. Mild reactions (grade I) were reported in this study, which could help patients and physicians investigate their cause to avoid anaphylactic reactions in future procedures. Once the investigation has been conducted, patients with a history of intra-operative immediate systemic reaction can be advised regarding future exposure, thus reducing the risk of subsequent anaphylactic reactions 19.

In conclusion, the incidence of intra-operative anaphylaxis observed in the present study was higher than previously reported incidences in other countries. This result should prompt allergists and anesthesiologists to establish systematic reporting of cases of intra-operative immediate reactions and implement preventive measures to reduce its occurrence.

## AUTHOR CONTRIBUTIONS

Garro LS, Soares IS, Motta AA, Kalil J, Carmona MJ and Giavina-Bianchi P conceived and designed the study. Garro LS, Aun MV, Soares IS and Ribeiro MR were responsible for acquisition of data. Garro LS, Soares IS, Aun MV, Kalil J, Castells MC, Carmona MJ, Giavina-Bianchi P performed analysis and interpretation of data. Garro LS, Ribeiro MR, Aun MV and Giavina-Bianchi P drafted the manuscript. Soares IS, Motta AA, Kalil J, Castells MC and Carmona MJ critically revised the manuscript for important intellectual content. All authors approved the manuscript final version to be published. All authors contributed to the drafting and revision of the manuscript. Finally, all authors have approved the version to be published and concur to this submission.

## Figures and Tables

**Table 1:** Types of surgical procedures during which intra-operative immediate hypersensitivity reactions occurred.

Type of procedure	n (%)
Non-vascular abdominal surgery	13 (21.7)
Plastic surgery	10 (16.7)
Ophthalmological surgery	10 (16.7)
Urological surgery	9 (15)
Otorhinolaryngological surgery	6 (10)
Gynecological/obstetric surgery	5 (8.3)
Neurosurgery	2 (3.4)
Thoracic surgery	1 (1.7)
Vascular surgery	1 (1.7)
Orthopedic surgery	1 (1.7)
Double surgical procedures	2 (3.4)
Total	60 (100)

**Table 2:** Clinical manifestations of intra-operative hypersensitivity reactions.

System	Clinical sign(s)	n (%)
Cutaneous	Rash	45 (75)
	Urticaria	7 (11.7)
	Rash and urticaria	5 (8.3)
	Angioedema	2 (3.3)
	Itching	2 (3.3)
Respiratory	Bronchospasm, dyspnea, decreased oxygen saturation	4 (6.7)
Cardiovascular	Tachycardia	4 (6.6)
	Bradycardia	6 (10)
	Mild hypotension	6 (10)
	Cardiovascular shock	5 (8.3)
Gastrointestinal	Vomiting, nausea, diarrhea	1 (1.7)

**Table 3:** Possible causative agents according to reaction grade severity.

Drugs	Grade I	Grade II	Grade III
	n (%)	n (%)	n (%)
NMBAs	16 (26.7)	4 (6.6)	2 (3.3)
Hypnotics	14 (23.3)	1 (1.7)	1 (1.7)
Antibiotics	2 (3.3)	3 (5)	0 (0)
Opioids	3 (5)	0 (0)	0 (0)
Latex	1 (1.7)	1 (1.7)	1 (1.7)
NSAIDs	1 (1.7)	0 (0)	0 (0)
Other	2 (3.3)	0 (0)	3 (5)
Not answered	11 (18.3)	6 (10)	1 (1.7)

NMBAs: Neuromuscular blocking agents, NSAIDs: Non-steroidal anti-inflammatory drugs.

*p*>0.05 (Fisher’s exact test).
